# Regulatory SNPs: Altered Transcription Factor Binding Sites Implicated in Complex Traits and Diseases

**DOI:** 10.3390/ijms22126454

**Published:** 2021-06-16

**Authors:** Arina O. Degtyareva, Elena V. Antontseva, Tatiana I. Merkulova

**Affiliations:** 1 Department of Molecular Genetic, Institute of Cytology and Genetics, 630090 Novosibirsk, Russia; degtyareva_rso@mail.ru (A.O.D.); antontseva@bionet.nsc.ru (E.V.A.); 2 Department of Natural Sciences, Novosibirsk State University, 630090 Novosibirsk, Russia

**Keywords:** regulatory SNPs, transcription factor binding sites, gene expression, gene by gene studies, genome wide approaches

## Abstract

The vast majority of the genetic variants (mainly SNPs) associated with various human traits and diseases map to a noncoding part of the genome and are enriched in its regulatory compartment, suggesting that many causal variants may affect gene expression. The leading mechanism of action of these SNPs consists in the alterations in the transcription factor binding via creation or disruption of transcription factor binding sites (TFBSs) or some change in the affinity of these regulatory proteins to their cognate sites. In this review, we first focus on the history of the discovery of regulatory SNPs (rSNPs) and systematized description of the existing methodical approaches to their study. Then, we brief the recent comprehensive examples of rSNPs studied from the discovery of the changes in the TFBS sequence as a result of a nucleotide substitution to identification of its effect on the target gene expression and, eventually, to phenotype. We also describe state-of-the-art genome-wide approaches to identification of regulatory variants, including both making molecular sense of genome-wide association studies (GWAS) and the alternative approaches the primary goal of which is to determine the functionality of genetic variants. Among these approaches, special attention is paid to expression quantitative trait loci (eQTLs) analysis and the search for allele-specific events in RNA-seq (ASE events) as well as in ChIP-seq, DNase-seq, and ATAC-seq (ASB events) data.

## 1. Introduction

A central goal of human genetics is to understand how genetic variation leads to phenotypic differences and complex diseases. Recently, genome-wide association studies (GWAS) have detected over 70 thousand variants (mainly, single nucleotide polymorphisms, SNPs) associated with various human traits and diseases [1,2]. The vast majority of the genetic variants identified from GWAS map to the noncoding part of the genome and are enriched in regulatory regions (promoters, enhancers, etc.), suggesting that many causal variants may affect gene expression [3,4,5,6].

As is known, the regulatory regions of the genome represent clusters of the binding sites for sequence-specific transcription factors (TFs). There, the interplay between these TFs and their binding sites (*cis*-regulatory elements) as well as the interaction of TFs with one another and the coactivator and chromatin remodeling complexes orchestrate the dynamic and diverse genetic programs, thereby determining the tissue-specific gene expression, spatiotemporal specificity of gene activities during development, and the ability of genes to respond to different external signals [7,8,9,10,11,12]. Thus, thanks to the binding to their specific sites on DNA (transcription factor binding sites, TFBSs), TFs directly interpret the regulatory part of the genome, performing the first step in deciphering the DNA sequence [13,14,15]. Consequently, regulatory SNPs (rSNPs), that is, genetic variation within TFBSs that alters expression, play a central role in the phenotypic variation in complex traits, including the risk of developing a disease.

Starting from the 1990s, numerous studies have been performed focusing on the noncoding SNPs that perturb the TF binding and are associated with various pathologies. As has been shown, risk alleles can (i) destroy a binding site for a TF [16,17,18,19]; (ii) create a binding site for a TF [20,21,22]; or alter the binding affinities towards an increase [23,24,25] or a decrease [25,26,27,28]. In addition, several cases have been observed when a damage/destruction of a binding site for a TF leads to a concurrent formation of another/other TFBS(s) [19,29,30].

The advent of the NGS technologies gave a strong impetus to the development of functional genomics and application of its methods to the genome-wide search for rSNPs. Currently, various methods of functional genomics are used for both mass interpretation of GWAS data and independent genome-wide identification of regulatory variants. So far, expression quantitative trait locus (eQTL) mapping and identification of allele-specific expression (ASE) events utilizing analysis of RNA-seq data (actually, the largest available genome-wide dataset) are the major relevant methods. The search for allele-specific binding (ASB) events in the data of DNase-seq, ChIP-seq, ATAC-seq (assay for transposase-accessible chromatin with high-throughput sequencing), and so on becomes ever more important. In addition, the approaches not directly associated with obtaining genome-wide data are actively used, including massively parallel reporter assay (MPRA), SNPs-seq, and SNPs-SELEX.

In this review, we brief the history of rSNP discovery, systematize and discuss the methods used in the studies of individual rSNPs, illustrate the narration with the case studies of several best-characterized rSNPs associated with different pathologies, and summarize the recent published data on the genome-wide approaches to the discovery and study of rSNPs.

## 2. Brief History of rSNP Discovery

The history of the research into the polymorphisms residing in noncoding gene regions and potentially able to influence the level of gene expression commenced as early as the 1990s. The SNPs associated with various pathologies were the main objects in this area. The medical genetic research at that time mainly focused on the variants localized to the gene coding regions [31]. Correspondingly, these studies were rather few [32,33] and the search for noncoding variants was frequently initiated by the absence of any SNPs associated with a disease in the coding part of candidate genes [30,34,35].

In particular, Comings et al. when studying the *TDO2* gene, a candidate gene in psychiatric genetics, failed to find any polymorphisms associated with psychiatric disorders in its coding part [36]. However, such variants were detected in intron 6, where a binding site for the receptor of glucocorticoids, the hormones stimulating *TDO2* expression, had been earlier identified [37]. According to Comings et al., both G→A and G→T substitutions, located 2 bp apart in the middle of intron 6, showed a significant positive association with drug dependence, Tourette syndrome, and attention deficit hyperactivity disorder [36]. Computer analysis emanating from conformational and physicochemical properties of TFBSs [38] predicted that both substitutions damage the binding site for Yin Yang 1 (YY1)—a transcription factor ubiquitously expressed throughout mammalian cells, regulating both transcriptional activation and repression and having a role in 3D chromatin organization [39]. For experimental confirmation the electrophoretic mobility shift assay (EMSA)—the method based on slower migration of protein–DNA complexes than free DNA fragments in gel electrophoresis—was used. EMSA with anti-YY1 antibodies confirmed the predictions made by showing the disappearance of the corresponding band in electropherogram due to preventing YY1 binding to DNA [30]. EMSA with specific antibodies demonstrated that both substitutions damaged the YY1 binding site and concurrently formed the binding sites for other unidentified TFs [30]. In another case, no mutations were detectable in the coding part of the candidate *CFTR* gene of several cystic fibrosis patients; correspondingly, its promoter region was examined and a T to G substitution was found at position −741 bp from the cap site, residing within a potential AP-1 binding site. Competitive EMSA demonstrated a change in the binding pattern of nuclear proteins resulting from this substitution but did not confirm any presence of AP-1 site [34]. In addition, no mutations in the coding part of the *GpIbβ* candidate gene were found in a patient with Bernard–Soulier syndrome; however, a C to G transversion at position –133 bp was detected in the 5′-upstream region of this gene. This changed a GATA consensus binding site, disrupted GATA1 binding (EMSA + antibody to GATA1), and decreased the promoter activity by 84% (CAT reporter assay) [35].

Other important examples of the rSNPs described at this time include a G to A substitution at −376 bp with respect to the *TNF* transcriptional start site; this substitution causes transcription factor OCT-1 binding (EMSA, ultraviolet crosslinking experiments, and specific antibodies) and alters the gene expression in human monocytes. As has been shown, the OCT-1 binding genotype is associated with a fourfold increased susceptibility to cerebral malaria in West and East African populations [40]; an A/G base transition within the Alu element preceding the *MPO* gene, associated with acute myelocytic leukemias, which creates a strong SP1 binding site (similarity to the consensus and EMSA with purified human SP1) and activates *MPO* transcription (CAT reporter assay) [41]; a G to A substitution detected at position 69 bp downstream of the polyadenylation site of delta-globin gene in a Northern Sardinian family affected by thalassemia. This substitution increases the GATA1 binding (EMSA + antibody to GATA1) and the authors believe it is responsible for a deficient function of the gene in question [42]. See the review by Deplancke et al. [32] for several other relevant examples.

Analysis of these papers demonstrates that the toolkit for rSNP studies was rather poor at that time, with gel shift experiments and transient transfection assays being the main used experimental approaches. As for bioinformatics search for the TFBSs with the structure changed by a nucleotide substitution, the consensus sequences deduced by that time or position weight matrices (PWMs) from TRANSFAC [43] were the main available approaches and an rSNP study in most cases ended in detection of a change in the sequence of a putative site.

However, the situation has radically changed since then. First, it has become clear that rSNPs play a leading role in the phenotypic diversity, in particular, to a considerable degree determining the susceptibility/resistance to diseases and individual sensitivity to various environmental factors, drugs included [4,5,32,44,45,46,47,48,49,50]. Second, the methodology for studying individual rSNPs has considerably expanded. Third, the advance in NGS technologies has formed the background for the approaches that allow the regulatory polymorphisms to be searched for on a genome-wide scale.

## 3. Modern Array of Methods for Studying Individual rSNPs

The two main methods mentioned above dating back to the beginning of the history of rSNP research—EMSA and reporter gene assay—still remain a golden standard in this area and are widely used in the state-of-the-art studies at the first stage of analysis because they allow the presence of a regulatory potential of a nucleotide substitution to be asserted. However, the expressed sets of TFs in different tissues are significantly different [11]; correspondingly, it is most desirable in such experiments to use several cell lines [3,23,51]. It is especially important that the EMSA with specific antibodies or purified TFs is able to reliably identify the TF with its binding site affected by a nucleotide substitution [23,28,30,40,41] and numerous other papers (Table 1). In a similar manner, such TF can be identified, although somewhat less unambiguously, in the reporter assays with cotransfection by the plasmids expressing suspected TFs [52,53,54].

However, EMSA, the most popular approach, is a strictly in vitro technique. As for verification of an in vivo TF binding to a region, ChIP-PCR is currently used [16,23,26,55,56], as well as its modification, ChIP-AS-qPCR. This modification allows the effect of a nucleotide substitution on a TF binding efficiency in a living cell to be demonstrated [20,24,57,58,59]. It is noteworthy that the identification of a TF with the help of listed methods requires the prior knowledge about the TFBSs harboring SNPs and this knowledge is usually acquired by bioinformatics analysis of the corresponding DNA sequence. Currently, different models of TFBSs, specialized databases, and the related tools are widely used for TFBS prediction, functional annotation of sequence variants, and prediction of the SNP impact on TF binding [60,61,62,63,64,65,66] and others. Moreover, the closer the result of bioinformatics analysis to the truth, the fewer labor and funds spent on the corresponding experiments. However, the tools currently used for TFBS discovery mainly rely on the recognition model of a traditional PWM [67]; this matrix is based on the hypothesis of additivity of different positions within TFBSs. This leads to considerable oversimplification of the mechanisms underlying the TF–DNA interaction and worsens the TFBS recognition efficiency [66,68,69,70].

Correspondingly, unbiased approaches to identification of the TFs with the binding sites affected by a nucleotide substitution are now developed. The most popular of them are (i) proteome-wide analysis based on the interaction of oligonucleotides corresponding to the alternative alleles with metabolically labeled nuclear factors followed by quantitative mass spectrometry [24,26]; (ii) oligonucleotide pull-down assay with subsequent mass spectrometric analysis [71,72]; (iii) mass spectrometric analysis of EMSA protein–DNA complex bands [73]. However, the same peptides can be present in different proteins (especially, in the TFs of the same family); thus, it is necessary to supplement this approach with additional experiments providing more precise data (for example, EMSA with specific antibodies [23,24] or immunoblotting [26]).

The effect of the already identified TFs with their binding sites affected by a nucleotide substitution on the expression of putative target genes is confirmed by siRNA-induced knockdown of the TFs [23,26,59] and/or their overexpression [16,20,26,74]. The same approaches are applied to detect widespread effects of rSNPs at the level of transcriptome [20,75].

In order to clarify whether the intergenic region carrying the target rSNP is potentially regulatory, the data of ENCODE projects [76] are usually assayed for the presence of DNase I hypersensitive sites (DHSs) and ChIP-seq peaks for active histone marks and transcription factors. If ChIP-seq peaks are numerous, this suggests a potential enhancer function, which is further verified by an increase in the luciferase reporter activity with recording of allele-specific effects [23,26,77,78]. To find out which particular genes are influenced by the studied region, it is inactivated using siRNA-mediated transcriptional gene silencing [23] or CRISPR interference (CRISPRi) involving recruitment of a KRAB repressor domain fused to catalytically dead Cas9 [18,79] or just via deletion of this region with CRISPR/Cas9 technology [28,78,80]. Then the transcription levels of the selected genes are assayed [23,28,79,80] or a whole transcriptome analysis is performed [78,80]. A physical interaction between the region carrying an SNP and the potential target genes is usually confirmed with the help of Hi-C methods [26,28,59,80], including allele-specific chromosome conformation capture assays [74], or using available Hi-C data [59,79,81].

CRISPR/Cas9-mediated single nucleotide editing becomes ever more popular when it is necessary to find out a direct effect of single base substitutions on the target gene expression [16,20,58,82,83] as well as widespread transcriptomic changes [80].

Recently, the toolkit for rSNP studies has been supplemented with assessment of the allelic expression imbalance (AEI or ASE) of the transcribed SNP, which either is regarded as a regulatory polymorphism [72,84] or is a marker for the rSNPs located beyond the transcribed genome part [23,55,56]. Either available heterozygous cell lines [23,72,84] or the heterozygous cells generated via CRISPR editing [84] are commonly used. Other recently used options are cells of healthy volunteers [24], biopsy specimens, or samples derived from patients during surgery [53,55,56,84]. This is an important advantage because a studied rSNP in this case exhibits its functionality under the conditions most close to the body’s natural context. Blood cells of healthy volunteers [85] and clinical samples [18,28,77,86] are also used to analyze the allele-dependent expression of individual genes by comparing the expression levels observed in the carriers of different genotypes. However, this kind of study requires a considerably larger number of participants as compared with ASE analysis.

## 4. Recent Comprehensive Examples

Here, we will describe the recent comprehensive examples of rSNPs associated with diseases (Table 2).

### 4.1. Allele C of rs36115365 from chr5p15.33 Multi-Cancer Risk Locus Enhances ZNF148 Binding and Telomerase Reverse Transcriptase (TERT) Expression

The rs36115365 polymorphism is one of the nine highly correlated SNPs residing in chr5p15.33 region 2 of the GWAS mapped multi-cancer risk locus. The performed EMSA functional analysis of these nine SNPs has shown that only the rs36115365 polymorphism displays the changes in protein binding pattern in EMSA with nuclear extracts of eight human cell lines [23]. (Note that its minor C allele of which is associated with an increased pancreatic and testicular cancer risk but a decreased lung cancer and melanoma risk.) This polymorphism is located in the region between the 5′ end of *TERT* (~18 kb upstream) and 3′ end of *CLPTM1L* (~5 kb downstream) genes. According to ENCODE data, this region is a putative enhancer since it overlaps with the multiple active histone modifications and TF ChIP-seq peaks. When transfecting the same eight cell lines, a 240-bp fragment carrying rs36115365 displayed an increase in luciferase reporter activity. In addition, allele C exhibited both preferred protein binding in EMSA and enhanced regulatory activity in reporter assay [23].

In order to clarify which of the neighboring genes are affected by the found enhancer, it was inactivated using siRNA-mediated transcriptional gene silencing [87]. This decreased the expression of *TERT* gene alone. The product of this gene, telomerase reverse transcriptase, in combination with an RNA template adds nucleotide repeats to chromosome ends, which is important to viability of cancer cells. A higher level of *TERT* expression from allele C was demonstrated using a marker SNP in the transcribed *TERT* gene part [23].

The TF the binding site of which changes as a result of a G to C substitution (rs36115365) was identified using the pull-down of nuclear proteins with the oligonucleotides corresponding to G or C allele followed by quantitative mass spectrometry [88]. Since four proteins that preferred allele C (ZNF148, VEZF1/ZNF161, ZNF281, and ZNF740) were detected, their binding was tested with EMSA: only the antibody to ZNF148 consistently caused a loss in the C allele binding. Moreover, the EMSA with recombinant purified ZNF148 confirmed specific binding to the C allele. Finally, siRNA-mediated knockdown of ZNF148 mRNA resulted in reduced *TERT* expression, telomerase activity, and telomere length. Thus, the C allele improves ZNF148 binding site, which elevates the *TERT* expression level and, as a consequence, increases the risk of multiple cancer types [23].

### 4.2. Allele G of rs11672691 from Chr19q13.2, Associated with Aggressive Prostate Cancer, Creates a HOXA2 Binding Site and Raises the Transcription Levels of PCAT19 and CEACAM21 Genes, Implicated in Prostate Cancer Cell Growth and Tumor Progression

The G allele of rs11672691 was identified by GWAS and additionally confirmed as being associated with aggressive prostate cancer by meta-analysis and genotyping of cancer cases and controls from 26 studies from European populations [20,89]. An eQTL analysis of The Cancer Genome Atlas (TCGA; Cancer Genome Atlas Research Network [90]) data, comprising about 1000 prostate tissue sample, has shown that the presence of allele G correlates with elevated transcription levels of the *PCAT19* and *CEACAM21* genes, both involved in the prostate cancer cell growth and tumor progression [20].

The rs11672691 polymorphism resides in the intron 2 of long noncoding RNA (lncRNA) PCAT19 and is 100 kb away from CEACAM21. The region housing this polymorphism is enriched in active enhancer marks, contains several TF peaks (ENCODE), and exhibits an enhancer activity in the luciferase reporter assay [20]. As is demonstrated using PWM, the rs11672691 polymorphism maps within the binding motifs of homeodomain transcription factors, including NKX3.1 and HOXA2; further, ChIP-AS-qPCR has shown that HOXA2 prevalently binds to allele G. HOXA2 knockdown decreases both PCAT19 and CEACAM21 expression. CRISPR/Cas9-mediated introduction of single nucleotide mutation was used to directly demonstrate that the presence of allele G led to higher levels of PCAT19 and CEACAM21 transcripts [91]; the genotype of rs11672691 was successfully converted from G/A to G/G or A/A in prostate cancer cell line 22Rv1. A comparison of the mutated and parental cells suggested that the G/G genotype was associated with higher transcriptional levels of PCAT19 and CEACAM21 as compared with the G/A and A/A genotypes; note that the transcriptional levels of these genes were the lowest for the A/A variant [20]. 

### 4.3. Atherosclerosis Risk Variant A of rs2107595 from Chr7p21.1 Interferes with E2F3 in Putative Enhancer Region, Which Leads to HDAC9 Activation

The rs2107595 polymorphism was identified by recent GWAS as the lead SNP for stroke and coronary artery disease (CAD) [92,93]. There are also numerous data indicating its involvement in the control of systolic blood pressure [94,95,96]. It resides in noncoding DNA 3’ to the HDAC9 gene in a region overlapping with DHSs and the histone activation marks H3K27ac and H3K4me1 (ENCODE). The search for the TF with ASB at rs2107595 commenced with the proteome-wide analysis of the interaction between the oligonucleotides that carried either risk (A) or normal (G) allele with labeled nuclear factors and subsequent quantitative mass spectrometry [88]. All constituents of the E2F3/TFDP1 (transcription factor Dp-1)/Rb1 complex were identified among the factors that prevalently bound to the G-centered oligonucleotide. The subsequent oligonucleotide pull-down assay followed by immunoblotting confirmed enriched binding of E2F3 to the common allele [26]. These findings agreed well with the presence of an E2F3 consensus sequence in the allele G region disrupted by the risk allele A [62]. ChIP-PCR in the HeLa cells homozygous for the G allele confirmed E2F3 binding in vivo. As was demonstrated, the rs2107595 risk A allele displayed a higher transcriptional capacity in luciferase assays as compared with common G allele and caused an increase in the *HDAC9* mRNA in genome-edited Jurkat cells [26]. Analysis of the allele-dependent expression of *HDAC9* in peripheral blood mononuclear cells of healthy donors also demonstrated increased mRNA levels of *HDAC9* only in risk allele carriers [85]. Since rs2107595 is located at a considerable distance from *HDAC9,* Prestel et al. [26] performed circularized chromosome conformation capture experiment [97] and discovered a physical interaction between rs2107595 and the *HDAC9* promoter in common allele (GG) but not in the risk allele cells (AA). This demonstrates the role of E2F3 in allele specific differences in the chromatin organization. These results suggest that an elevated *HDAC9* expression is involved in the etiology of stroke and CAD and that *HDAC9* targeted inhibition is one of the strategies to prevent atherosclerosis although the mechanism underlying a promoting effect of increased *HDAC9* expression on atherogenesis and vascular risk is vague [26].

### 4.4. Allele A of rs12411216 from Chr1q22 Decreases E2F4 Binding, Which Results in a Decreased GBA Expression and an Increased Cognitive Damage in Parkinson’s Disease

As is known, a decrease in the glucocerebrosidase (*GBA*) gene expression in the brain promotes a prion-like spread of α-Syn interpolymer complexes and progression of Parkinson’s disease (PD) as well as increases the cognitive damage [98,99]. Jiang et al. [28] were the first to identify the rs12411216 polymorphism as an eQTL that influences the *GBA* gene expression. Genotyping of 122 PD patients with mild cognitive impairment (PD-MCI) and 184 PD patients who had PD but no cognitive impairment suggested a statistically significant correlation between the A allele and PD-MCI. In addition, the *GBA* mRNA level was significantly decreased in clinical samples of the patients with AA genotype as compared with the patients with CC. The rs12411216 polymorphism is located at a distance of ~50 kb from the *GBA* transcriptional start site and falls into one of the DHSs that contacts the *GBA* promoter as is suggested by Hi-C data. According to the TFBS database (wgEncodeRegTfbsClustered), rs12411216 overlaps with the core motif of E2F4 TF, which was confirmed by EMSA with specific antibodies. EMSA has also shown a drastic decrease in the E2F4 binding in the case of the risk A allele. A CRISPR/Cas9-mediated deletion of the DHS housing rs12411216 decreased *GBA* expression, weakened the enzyme activity, and enhanced an abnormal aggregation of α-Syn in SH-SY5Y cells [28]. Interestingly, a little earlier a significant association of rs12411216 with occipital lobe volume in the European ancestry-only meta-analysis was found [100]. The authors also showed that the most significant genetic correlation with brain lobar volume and diseases was observed between occipital lobe volume and Parkinson’s disease (rg = 0.18, *p* = 0.03). However, this finding was not significant after multiple testing correction, which makes the authors consider it as a preliminary result [100].

### 4.5. Allele A of rs13239597, Associated with Two Systemic Autoimmune Diseases, Enhances the Binding of EVI1, Which Promotes Formation of a Long-Range Chromatin Loop and an Increased Expression of IRF5, Located 118 kb Away

GWAS identified genetic variants conferring the risks of autoimmune diseases systemic lupus erythematosus (SLE) and systemic sclerosis (SSc) at 7q32.1, harboring *IRF5* and *TNPO3* genes. The rs13239597 polymorphism is located in the *TNPO3* promoter 118 kb away from *IRF5.* According to eQTL analysis involving 373 unrelated European samples of lymphoblastoid cell lines, the minor allele A of rs13239597 was significantly associated with an increased *IRF5* expression; this was also confirmed with GTEx data [6]. On the other hand, any significant association between rs13239597 and *TNPO3* was unobservable. Analysis of the available Hi-C data demonstrates that *IRF5* is among the 12 genes that interact with rs13239597. A motif analysis predicted four potential TFs with ASB affinity to rs13239597, namely, EVI1, ERF, GATA1, and TAL1. In order to find out which particular TF influences the *IRF5* expression, their shRNA-mediated knockdown in U2OS cell line was performed. A significant decline in the *IRF5* expression was detected only in EVI1 knockdown U2OS cells. Moreover, a 3C assay showed that EVI1 knockdown significantly decreased the interaction between rs13239597 and *IRF5* promoter. Then, ChIP-AS-qPCR demonstrated that EVI1 was preferentially recruited to the rs13239597 A allele as compared with its C allele.

Finally, analysis of three SLE genome-wide gene expression datasets revealed a significantly higher *IRF5* expression in the SLE patients as compared with healthy subjects [59].

### 4.6. Allele T of rs17079281 Decreases Lung Cancer Risk through Creating an YY1 Binding Site to Suppress Proto-Oncogene DCBLD1 Expression

According to GWAS, rs9387478 in 6q22.2 is associated with lung cancer risk in both Asian [101] and European populations [102]. Linkage disequilibrium (LD) analysis, meta-analysis involving 4403 cases and 5336 controls, and two additional case–control studies have discovered a novel SNP, rs17079281, in the *DCBLD1* promoter, which is associated with lung cancer risk in Chinese populations [16]. As is shown, the patients with T allele have a lower risk of adenocarcinoma as compared with the carriers of C allele (adjusted OR = 0.86; 95% CI: 0.80–0.92) and that the subjects with the C/T or T/T genotype have lower levels of *DCBLD1* expression than those with C/C genotype in lung adenocarcinoma tissues [16]. According to TRANSFAC data [43], a C→T substitution in this region may create an YY1 binding site. This is confirmed with the help of ChIP-qPCR analysis in wild-type Beas2B cells (C/T at rs17079281) and the CRISPR/Cas9 modified cells with C/C knockin, which demonstrated that T allele was necessary for binding YY1. Transfection of these lines with a plasmid expressing this TF showed a decrease in the *DCBLD1* expression only in wild-type Beas2B cells. Thus, the YY1 transcription repressor has a higher binding affinity for the T allele of rs17079281, which results in suppression of *DCBLD1* proto-oncogene expression and, consequently, in a decreased lung adenocarcinoma risk [16].

## 5. rSNPs on a Genome-Wide Scale

**Figure 1:**
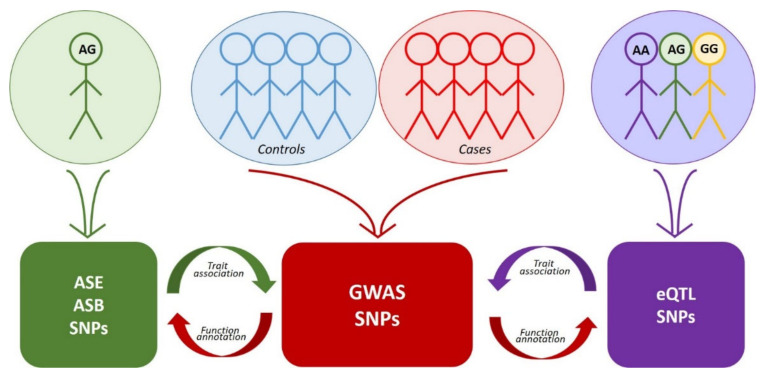
Interplay between the approaches to the search for functional SNPs. Colored blocks denote arrays of corresponding data. Red arrows indicate functional annotation of GWAS data using eQTL, ASE or ASB analysis. Purple arrow indicates the search for association of eQTL SNPs with traits via comparison with GWAS data. Green arrow—the same for SNPs detected by ASE or ASB analysis.

Genome-wide approaches to the search for rSNPs fall into two large groups. The first group comprises GWAS mass data analysis utilizing manifold methods of functional genomics, while the second group uses the same methods but independently without any prior knowledge about trait associations (Figure 1, Table 3). The latter group includes eQTL analysis, identification of allele-specific events, and some other genome-wide approaches. As for the rSNPs discovered by the approaches of the second group, it is necessary to additionally determine their association with a certain trait (most frequently, via comparison with GWAS data or by analysis of rSNPs as an eQTL in transcriptome data and reconstruction of the gene networks and molecular pathways).

### 5.1. Making Molecular Sense of GWAS

Historically, GWAS is the first genome-wide approach to identification of the genetic variants (mainly SNPs) associated with traits. Having appeared in the mid-2000s, GWAS have so far detected over 70 thousand loci associated with various human traits and diseases [1,2]. However, this technology is unable to give any information about the functionality of discovered variants, making it very difficult to translate GWAS data into biological insights, which is necessary to reveal the molecular mechanisms underlying diseases [25,103]. In addition, GWAS cannot distinguish between causal polymorphisms and numerous marker SNPs detected due to LD. Thus, considerable efforts have been recently focused on the subsequent functional analysis of the SNPs with disease/trait associations revealed with the help of GWAS. Both individual SNPs (mapped by GWAS and according to LD) [16,18,20,23,26,53,74,78,82,104] and others and large arrays of polymorphisms are analyzed in this way; manifold methods of the state-of-the-art functional genomics are used for this purpose.

One of the approaches in functional genomics frequently applied to mass functional interpretation of the SNPs detected by GWAS is MPRA in different variants [24,79,84,105,106]. MPRA is an upscaled version of gene reporter assay allowing the effect of an allele on the expression of a reporter construct to be determined with concurrent testing of several hundred to several thousand DNA fragments [107]. In particular, this method was used to test 1605 SNPs, 35 of which were associated with osteoarthritis in Europeans via GWAS and the remaining ones were in LD with them [84]. Six of these polymorphisms displayed differential regulatory activity between the major and minor alleles in the STARR-seq MPRA in Saos-2 osteosarcoma cell line and for three of them, this activity was confirmed by conventional luciferase reporter system. A more detailed study of the most significant SNP, rs4730222, showed differential nuclear protein binding in EMSA as well as the effect of alleles on the expression level of HBP1 isoform, transcribed from an alternative promoter containing rs4730222 at position +80 bp relative to its transcriptional start site [84]. Analogously, MPRA was used to study 832 variants associated with melanoma risk; 30 of them displayed significant difference between two alleles in UACC903 melanoma cells [24]. The rs398206 polymorphism, located in the first intron of *MX2* gene, was studied in detail; a most pronounced allelic difference was observed. As was shown, the risk-associated A allele significantly increases the YY1 binding to a DNA region carrying rs398206 in vitro (EMSA) and in vivo (ChIP-AS-qPCR), leading to an increase in *MX2* expression, which accelerates melanoma formation [24]. Analysis of these data suggests that the number of MPRA-revealed rSNPs is relatively small (0.1–4.7% of the tested GWAS variants). Perhaps, this is explainable in part with the use of only one cell line in each case. Due to tissue-specific effects, the use of several cell lines could increase the number of SNPs displaying significant difference between two alleles.

Similar to MPRA as an upscaled variant of gene reporter assay, an upscaled EMSA variant—Reel-seq (Regulatory element-sequencing)—was designed [25]. For this approach, a sequence library containing disease-associated SNP constructs with both the risk and non-risk alleles were generated by massive parallel oligonucleotide synthesis. After binding to nuclear proteins from MDA-MB-468 cell line and several EMSA rounds, the SNPs that demonstrated allele-imbalanced gel shift pattern between the risk and non-risk allele rSNPs were selected from this library [25]. Thus, 521 (12%) potential rSNPs were selected out of 4316 breast cancer-associated (GWAS) SNPs. Allele-specific effects were confirmed for 12 of the selected polymorphisms by conventional EMSA and luciferase assay. For three SNPs from breast cancer-associated FGFR2 locus, the TFs with the binding altered as a result of an SNP were identified using the approached devised by the authors: SNP-specific DNA competition pulldown-mass spectrometry (SDCP-MS) and allele-imbalanced DNA pulldown–Western blot (AIDP-Wb). Thus, the authors succeeded in demonstrating that the TFs PARP-2 and TFAM bound to rs7895676 with less binding of risk allele C; TFs TEAD1 and TEAD3 bound to rs2981578 with more binding of risk allele G; and NFIB bound to rs2981584 with less binding of risk allele G.

In its essence, Reel-seq is similar to the earlier described SNPs-seq [108], which differs from it only by the method used to distinguish between the protein-bound DNA oligonucleotides from free oligonucleotides (for this purpose, a protein purification column is used in SNPs-seq). SNPs-seq has been used to study allele-dependent protein binding at 903 SNPs identified by GWAS as variants that increase prostate cancer risk. Using the nuclear extract of prostate adenocarcinoma cell line LNCaP, 403 SNPs (45%) that showed protein-binding differences (>1.5-fold) between the reference and variant alleles were found. Of interest is that the rate (percentage) of the detected functional SNPs in GWAS data using Reel-seq and SNPs-seq methods was by an order of magnitude higher as compared with MPRA despite that only one cell line was used. This fact is explainable with a very high regulatory potential of naked DNA, discovered in our earlier studies on computational recognition of TFBS and experimental verification of the predicted sites by EMSA [109]. In reporter studies, several factors can conceal this effect, such as the TFBS position relative to promoter and the need in target TF interaction with both close and remote partner TFs.

The year of 2021 brought about another high-throughput method for assessment of the direct effect of a nucleotide substitution on TF binding—SNP-SELEX—an ultra-high-throughput multiplex protein–DNA binding assay [66]. SNP-SELEX utilizes a library of 40-bp DNA sequences matching the reference human genomic sequence in which the tested SNP permutated to all four bases located in the center. In the Yan et al. [66] study, the library consisted of 383544 distinct oligonucleotides corresponding to 95886 SNPs, including those linked to T2D susceptibility via GWAS and those located in putative *cis*-regulatory sequences 500 kb of T2D-tagging SNPs. Using 270 recombinant human TFs, the authors performed 828 million measurements of transcription factor–DNA interactions and succeeded in discovering 11079 SNPs (11.5% of the analyzed ones) that exhibited significantly differential binding to at least one TF [66].

Functional genomics data available in the current databases are also widely used in a high-throughput interpretation of GWAS data. In particular, Li et al. [110] used the ENCODE ChIP-seq data for 34 TFs obtained using human brain tissues or neuronal cells and bioinformatics search (using PWM) for TFBSs in ChIP-seq peaks. Analysis of the 8005 SNPs (including 40 index SNPs and those that were in LD with index SNPs) associated with major depressive disorder (MDD) [111] detected 34 MDD risk SNPs that disrupted the binding sites for 15 TFs. The allelic effect on reporter gene expression was confirmed for 29 of the analyzed SNPs, one of these polymorphisms, rs3101339, appeared to be associated with the *NEGR1* gene in qQTL analysis as well as affected its expression in the experiments on knockout of the region containing rs2050033 using CRIPSR-Cas9-mediated genome editing. This suggests that rs3101339 may confer MDD risk by affecting *NEGR1* expression [110]. The ChIP-seq datasets for various histone modifications were used to construct a comprehensive list of super enhancers in T2D [112] and CAD [113]. The rVarBase [114] was used in further functional annotation of super enhancer SNPs. This gave 286 T2D- and 366 CAD-associated super enhancer SNPs, part of which was annotated as being involved the regulation of chromatin structure and in the effects on TF binding [112,113]. Similarly, the own ChIP-seq data for the histone modifications marking the active regulatory elements of the genome were used for analyzing the GWAS SNPs associated with risk of epithelial ovarian cancer [115]. H3K27Ac ChIP-seq were generated for 26 ovarian cancer and precursor-related cell and tissue types and in combination with motifbreakR tool allowed for the discovery of 469 candidate causal risk variants in H3K27Ac peaks that were predicted to significantly break TF binding motifs [115].

There are many other examples of a global functional interpretation of the SNPs from the GWAS Catalog with the use of the data on TF-based motifs, promoters, enhancers, chromatin accessibility landscapes, three-dimensional chromatin interactions, and, especially, eQTL analysis [19,116,117,118,119,120,121,122].

### 5.2. eQTL Analysis

eQTL mapping is used to identify the association of a genetic variant with gene expression level based on transcriptome analysis. The term eQTL either means the presence of such association between a variant (eVariant) and the expression level(s) of gene/genes (eGene/eGenes) [6,123,124] or refers to the variant itself that displays such association [28,125,126,127,128]. When searching for an eQTL, differential gene expression in the transcriptomes of the subjects with different genotypes is determined for each SNP. Unlike GWAS, requiring tens and hundreds of thousands of participants, eQTL mapping requires just several hundreds of samples [6,124,125]. Transcriptome data alone are sufficient to detect the eVariants located in the transcribed region [129], while detection of all eQTLs also demands whole genome sequencing data [124].

Initially, eQTL analysis was conducted using microarrays and later, RNA-seq was used. Note that the very first studies demonstrated that eQTL effects considerably varied between the examined cell types or tissues (see review [130] for numerous examples). That is why the Genotype-Tissue Expression (GTEx) project was initiated in 2010 with the goal to create the catalog of events of the effects of genetic variants on gene expression determined in the maximally possible number of human tissues. The goal was to eventually detect the association of such variants with complex diseases and traits and to get a deeper insight into the molecular mechanisms underlying their action [131].

Currently, the GTEx Consortium has at its disposal the results of analysis of 15,201 RNA-seq samples from 49 tissues of 838 postmortem donors. In total, 4,278,636 genetic variants (cis-eQTLs) associated with a change in expression level of 18,262 protein coding and 5006 lincRNA genes have been found; each of them manifested itself at least in one tissue. All this suggests the presence of regulatory associations for almost all genes in the human genome. The genes lacking a cis-eQTL have emerged to be mainly the genes that lack any expression in the analyzed tissues, in particular, the genes that are active only in the early development. In addition, the genome regulatory regions and GWAS loci have been shown enriched in eQTLs [124]. However, detection of the causal variants remains challenging in both eQTL analysis and GWAS because of the presence of multiple variants in LD [132,133].

At present, eQTL analysis is actively used to identify trait-associated genes, especially susceptibility genes from GWAS loci, since this informs on the genes for which expression levels correlate with trait-associated variants [134,135,136,137,138,139,140,141,142,143]. Although eQTL analysis is not initially associated with any prior knowledge about traits, the data on differential gene expression in the individuals with different genotypes obtained with this method are also helpful in detection of the functional associations between these genes and construction of gene networks. This makes it possible to determine putative phenotypic outcomes for at least part of the detected eSNPs [71,134,135,144,145,146].

However, many eQTLs (eVariants) map to genome regulatory regions; correspondingly, the results of eQTL analysis are frequently the starting point in identification of rSNPs. In particular, rs12411216, the A allele of which decreases E2F4 binding and *GBA* gene transcription and thus increases the cognitive damage in PD, was first identified as an eQTL affecting *GBA* gene expression [28]. In addition, eQTL analysis has been used for prioritization of rs13239597, linked to lupus systemic erythematosus and systemic sclerosis via GWAS. Further studies of rs13239597 demonstrated that its risk A allele increased EVI1 binding and acted as an allele-specific enhancer regulating *IRF5* expression [59]. Both examples are detailed in Section 4 of this review. In a similar manner, the rs10085588 polymorphism, associated with bone mineral density and osteoporosis, was initially identified as an eQTL for *SLC25A13.* Its minor allele A displayed a decreased gene expression both in vivo in human primary osteoblasts and in vitro in luciferase reporter assay [86]. In addition, eQTL analysis detected six potential functional SNPs (rs9533090, rs9594738, r8001611, rs9533094, rs9533095, and rs9594759) exclusively correlated with the *RANKL* gene expression. They all belong to the group of multiple intergenic SNPs located over 100 kb upstream of the *RANKL* gene, associated with osteoporosis via GWAS. Later, one of these polymorphisms, rs9533090, was identified as an allele-specific regulatory SNP. The variant of the C allele resulted in the binding of TF NFIC, which led to the activation of enhancer and an increase in the expression of *RANKL,* a key regulator of bone metabolism [81]. Manifold methods of functional genomics, such as MPRA and identification of active chromatin modifications and open chromatin regions, are used in the mass search for rSNPs among eQTLs variants [147,148,149].

### 5.3. Allele-Specific Expression (ASE) Analysis

RNA-seq technology gives a brilliant opportunity of quantifying the expression of two alleles of any polymorphic sites in a diploid individual and of detecting allelic imbalance of transcription or an ASE event. In turn, ASE mapping is a useful instrument making it possible to identify variations in gene expression underlying phenotypic differences among individuals [126,133,150].

Typically, ASE events are detectable by joint analysis of transcriptome data and the WGS (whole genome sequencing) data for the same individuals. For example, Kang et al. [151] used RNA-seq data for lymphoblastoid cell lines derived from 77 unrelated European subjects (their genomic data are available through the 1000 Genomes Project) and discovered 2309 SNPs associated with ASE patterns. These SNPs were enriched in promoter regions and 108 of them had been earlier associated with human immune diseases [151]. Liu et al. [152] utilized RNA-seq and WGS data from a single cancer sample for each of the 13 pediatric T-lineage acute lymphoblastic leukemias (T-ALLs) and found dozens of somatic noncoding regulatory variants able to cause cis-activation of 222 candidate genes. These variants comprised both known noncoding mutations activating T-ALL oncogenes (*TAL1/2*, *LMO1/2,* and *TLX3*) and the new ones, including a C to T substitution in the *TAL1* intron 1, which created an YY1 binding site and, as a consequence, activated an enhancer residing in the same region [152]. RNA-seq and WGS data from the GTEx v8 release [124] allowed Castel et al. [150] to generate an ASE resource containing in total 431 million ASE events at an SNP level and 153 million measurements at a haplotype level. However, when genotype information is not available, it could be derived from RNA-seq reads directly via their sophisticated allele-specific analysis [46,144,153].

One of the main advantages of ASE approaches as compared with eQTL analysis and the others, GWAS consists in that both eQTL and GWAS rely on the analysis of numerous samples from the subjects with different genetic backgrounds and conditions of individual life. As for ASE approach, it relies on comparison of allelic effects within subjects and thus controls genetic background and cell environment; this allows the sample to be significantly reduced even to a single individual [153]. Thus, ASE approach gives the opportunity to considerably increase the number of temporal and environmental conditions that can be analyzed in parallel, thereby providing unique possibilities, first and foremost, for the studies in pharmacogenetics and pharmacogenomics. A perfect example here is the study by Moyerbrailean et al. [46] on detection of allele-specific effects of 50 substances (steroid and peptide hormones, metal ions, dietary components, common drugs, and environmental contaminants) using five types of cells (LCLs, PBMCs, HUVECs, SMCs, and melanocytes) each derived from three individuals. Analysis of transcriptome data allowed the authors to discover 1455 genes with ASE events and to identify 215 genes with gene-by-environment (GxE) interactions [46]. In a similar manner, condition-dependent ASE events in 19 genes related to the inflammatory response were detected via RNA-seq of primary white blood cells from eight human subjects before and after LPS treatment [154]. In addition, M0 and M1 macrophage states were compared using the samples of 48 healthy subjects. This gave 408 and 334 unique ASE events in MO and M1 state, respectively, while 1280 genes showed evidence of ASE under both conditions [126].

Gutierrez-Arcelus et al. [83] studied the enrichment dynamics of the alleles of heterozygous SNPs in transcriptomes during development of the response to an external stimulus. The authors analyzed their RNA-seq data for eight time points (0, 2, 4, 8, 12, 24, 48, and 72 h) during memory CD4+ T cell (from 24 genotyped individuals) activation by anti-CD3/CD28 beads. The result was 561 dynamic ASE events where the reference and alternative alleles demonstrated different patterns in time, including 182 dynASE events in MHC locus and 15 events in *HLA-DQB1.* Using CRISPR/Cas9 editing in the HLA class II expressing T cell line (HH), they demonstrated that the allele G of rs71542466, located 39 bp upstream of the *HLA-DQB1* transcription start site, increased its expression [83].

### 5.4. Allele-Specific Binding (ASB) Analysis

Although ASE analysis is most efficient for identifying gene expression variations, it yet fails to answer the question on whether the observed effect is a direct result of a nucleotide substitution in the SNPs used to measure ASE or simply captures the effects of other cis-acting variation. Our present consensus is that most of the disease-associated SNPs are located in regulatory regions [5]; there, they can lead to ASB of TFs with subsequent differential expression of the target gene alleles. Several studies focused on a genome-wide detection of ASB events have been so far completed. This direction commences from the pioneering research aimed at identification of the sequence variants that influence TF occupancy in the accessible chromatin (DNase-seq) [155] and TF ChIP-seq data [156].

Maurano et al. [155] have analyzed 493 high-resolution DNase-seq profiles (both published and acquired by the authors) from diverse cultured primary cells, cultured multipotent and pluripotent progenitor cells, and fetal tissues of 166 individuals and 114 cell types. In total, they succeeded in detecting 64,599 SNPs that displayed allelic imbalance in chromatin accessibility. Using PWMs for 2203 TF motifs from TRANSFAC [157], JASPAR [158], UniPROBE [159], and a published SELEX dataset [160], the authors demonstrated that the majority of the found SNPs are able to directly influence the TF occupancy and, as a consequence, to change regulatory DNA accessibility in vivo [155].

A direct genome-wide search for asymmetric TF binding events with the help of public ChIP-seq data was for the first time performed in Claes Wadelius’s laboratory. The authors have analyzed the TF ChIP-seq data available at the time of download for four cell lines—GM12878 (B cells), H1-hESC, K562, and SK-N-SH from ENCODE project—and discovered 9962 SNPs with biased TF allele binding. Their computations suggested that the most common polymorphisms could be tested for ASB via repeated ChIP-seq experiments with 20 selected TFs in 3–10 individuals [156]. Further analysis of their data showed that 141 of the detected AS-SNPs emerged to be associated with different GWAS traits (15 were listed in the GWAS catalog and 126 fell in a high-LD interval); 84 AS-SNPs detected in B cells coincided with eSNPs for B cells [161]; an additional 362 AS-SNPs were in LD with an eSNP [156]. The same approach allowed for detection of 3713 SNPs displaying significant difference in the binding between alleles in HepG2 and HeLa-S3 cell lines. The dual luciferase reporter assay of 39 of them confirmed ASE of 27 [162]. A comprehensive functional analysis of the rs953413 polymorphism, identified as an AS-SNP in human liver HepG2 cells and located in an evolutionarily conserved enhancer element in the first intron of *ELOVL2* gene [162], has shown that the A allele disrupts a FOXA binding site. This decreases the binding not only of this TF, but also of HNF4α, which cooperatively interacts with it; correspondingly, this leads to a decrease in *ELOVL2* expression and impaired hepatic docosahexaenoic acid synthesis, which may play a role in the pathogenesis of nonalcoholic fatty liver disease [58].

Recently, ATAC-seq [163] was applied in a genome-wide search for ASB events concurrently for all TFs functioning in the cell. ATAC-seq makes it possible not only to detect genomic footprints left by DNA-binding proteins, but also to determine the allelic bias in the binding of these proteins [164]. Using ATAC-seq, the authors succeeded in detecting 53 rSNPs in human MCF-7 breast cancer cells and 125 rSNPs in human mesenchymal stem cells (MSCs). Using their own RNA-seq data and publicly available chromatin interaction data for MCF-7 cells, they demonstrated that the detected 53 rSNPs were associated with 74 potential target genes. A comparison of rSNPs with the eQTLs from GTEx Project database demonstrates that 30% of the rSNPs from MCF-7 and 43% from MSC fall into eQTL regions, suggesting their role in the allelic differences in gene expression [164].

A genome-wide search for ASB events for the binding sites of an individual TF has been also performed. In particular, such search was conducted for NKX2–5, a cardiac-specific TF [165]; according to GWAS data, this TF can be regarded as a candidate gene associated with EKG phenotypes [166]. For this purpose, 15 ChIP-seq experiments with anti-NKX2–5 antibodies were performed in pluripotent stem cell-derived cardiomyocytes from seven related individuals. As a result, about 2000 SNPs with allele-specific effects on NKX2-5 binding were discovered; they were enriched for altered TF motifs, heart-specific eQTLs, and EKG GWAS signals [165]. Allele-specific effects of two of these SNPs (rs3807989 and rs590041) were confirmed by EMSA, luciferase assay, and analysis of their effect on target gene expression [165].

The ChIP-seq data for whole-genome histone modification profiles are also helpful in the search for ASB events on a genome-wide scale; these profiles characterize the energy landscape of chromatin, whereto the TF binding with regulatory regions considerably contributes [167,168,169,170]. In particular, this approach was implemented when searching for ASB events in K562, MCF-7, and HCT-116 human cell lines by analyzing the ENCODE ChIP-Seq data for histone epigenetic modifications (H3K27ac, H3K4me1, H3K4me2, H3K4me3, and H3K27me3) and 456 different chromatin-associated proteins, mainly transcriptional factors [171]. ASE events were also assessed in HCT-116, MCF-7, and K562 cells (ENCODE) using RNA-Seq and ChIA-PET data with an RNA pol II antibody. This allowed for detection of 1633 rSNPs simultaneously associated with both types of allele-specific events. According to GWAS data, 27 of them were associated with a risk of malignancy [171] and 14 with cognitive disorders [172]. In addition, an association with colorectal cancer (CRC) was suggested for 30 rSNPs based on a comparison of allele frequencies in the ICGC cohort [173] with the MAFs reported by dbSNP [171]. Genotyping of CRC patients and healthy controls according to six of these polymorphisms demonstrated that rs590352 of *ATXN7L3B* gene was associated with CRC in men and rs4796672 of *KRT15* gene, with CRC in women. In addition, the analysis of haplotypes shows that rs2072580, located in the promoter region common for the *ISCU* and *SART3* genes, can be also associated with CRC [174].

The allele-specific signals were also searched for in the ChIP-seq data for histone modifications in a study aimed to identify the regulatory variants involved in the development mechanisms of immune and B-cell related diseases [175]. The SNPs with allele-specific behavior in the available ChIP-seq datasets produced for the histone modifications defining promoters (H3K4me3) and enhancers (H3K4me1 and H3K27ac) and for domain boundary proteins (CTCF and SA.1) were the focus of this study. Thus, 17293 such SNPs (AS-SNPs) were found in seven lymphoblastoid cell lines; of them, 237 were associated with immune GWAS traits and 714 with gene expression in B cells.

## 6. Conclusions

Gene expression programs underlying development, differentiation, and environmental responses are guided by the regulatory DNA portion of the metazoan genomes. The corresponding information encoded in regulatory DNA is actuated via the combinatorial binding of sequence-specific TFs to regulatory regions (cis-regulatory modules, CRMs). CRMs switch on promoters and enhancers and are actually the assemblies of TFBSs arranged to provide particular functions [10,11,14,176,177,178].

The SNPs located in transcriptional regulatory regions can alter gene expression, which may be either adaptive or lead to a disease. The main mechanism underlying the action of these SNPs consists in changes of TF binding, which comprises creation or disruption of TFBSs (cis-regulatory elements) or alteration of the affinity of TFs for their cognate sites [32,155,179,180]. Although many SNPs with such properties have been so far discovered, their mass search in genomes remains challenging. This is mainly associated with the tissue, developmental, and environmental specificities in the effects of rSNPs, which is a direct consequence of the corresponding specificities of the harboring cis-regulatory elements [32,45,124]. Thus, myriads of omics experiments are necessary for this purpose; however, this is still too expensive and time-consuming. The computer methods for recognition of TFBSs in DNA sequences are free of this disadvantage but yet ineffective in detection of both TFBSs and the SNPs changing these sites without the cooperation with omics experiments. The objective reasons here are a high degeneracy of the regulatory DNA code [15,109,181,182]; high importance of low-affinity sites in gene regulation [183]; the presence of structural variants of the binding sites for the same TF [184,185,186,187]; and even nonconsensus TFBSs [188,189]. All these facts considerably decrease the efficacy of the available methods for TFBS recognition, most of which are based on the PWM model, which oversimplifies the mechanisms underlying TF–DNA interaction [66,68,69,70]. Development of new generation bioinformatics approaches relying on machine learning and neural networks raises the hope for more efficient and accurate recognition of both the TFBSs and rSNPs in the genomes [190,191,192,193,194,195].

Thus, despite the achieved progress, we are still at the beginning of the way to comprehensive annotation of the genome regulatory portion, full cataloging of rSNPs, and clarification of their association with molecular phenotypes and, eventually, with various complex traits, including diseases. The further advance requires improving the efficiency of the existing experimental and bioinformatics methods of systems biology and advent of the new relevant approaches.

## Figures and Tables

**Table 1:** Experimental methods for analysis of rSNPs.

Aim	Method	Advantages	Shortcomings	Comments
Registration of the fact of an effect of nucleotide substitution on TF binding	EMSA with nuclear extract (cross-competition assay when necessary)	Simple procedure	In vitro; tissue-specific effects	Testing of several cell lines is desirable
Identification of TF the binding site of which is disrupted by a nucleotide substitution	EMSA with purified TF or specific antibody	Unambiguous result	In vitro; requires prior knowledge about TFBS, purified TF, specific antibody	Prescreening in competition assay with unlabeled oligonucleotides may be helpful
Confirmation of TF binding in vivo	ChIP-PCR	In vivo	Requires prior knowledge about TFBS and specific antibody	
Identification of TF the binding site of which is disrupted by a nucleotide substitution	ChIP-AS-qPCR	In vivo; unambiguous result	Requires prior knowledge about TFBS and specific antibody	Copy number variation must be taken into account when using cell lines
Identification of TF the binding site of which is disrupted by a nucleotide substitution	Pull-down assay followed by mass spectrometry analysis	Requires no prior knowledge about TFBS	In vitro	Confirmation by EMSA with purified TF or specific antibody is necessary in some cases
Registration of the fact of an effect of nucleotide substitution on the activity of regulatory element	Reporter assays	Simple procedure	Out of genome context	Testing of several cell lines is desirable
Registration of the fact of an effect of nucleotide substitution on the activity of regulatory element	CRISPR/Cas9-mediated single nucleotide editing	In genome context		Testing of several cell lines is desirable

**Table 2:** Examples of rSNPs altering TFBSs and affecting gene expression.

ID	Location	Risk Allele	TFs with ASB	Genes with ASE	Risk Disease According to GWAS	Ref
rs36115365	chr5p15.33 intergenic region, putative enhancer	C	ZNF148 (EMSA+AB, EMSA+ purified ZNF148)	*TERT * (ASE, siRNA-mediated knockdown of ZNF148)	Increased pancreatic and testicular cancer risk but a decreased lung cancer and melanoma risk	[23]
rs11672691	Chr19q13.2 Intron 2 of lncRNA *PCAT19*	G	HOXA2 (ChIP-AS-qPCR)	*PCAT19* *CEACAM21* (ASE, HOXA2 knockdown CRISPR/Cas9	Aggressive prostate cancer	[20]
rs2107595	Chr7p21 noncoding DNA 3’ to the *HDAC,* DHSs	A	E2F3 (ChIP-PCR)	*HDAC9* (ASE)	Atherosclerosis, coronary artery disease, stroke	[26]
rs12411216	Chr1q22 DHSs	A	E2F4 (EMSA+AB)	*GBA* (ASE, CRISPR/Cas9)	Parkinson’s disease, cognitive damage	[28]
rs13239597	Chr7q32.1 *TNPO3* promoter	A	EVI1 (ChIP-AS-qPCR)	*IRF5* (ASE, shRNA-mediated knockdown of EVI1)	Systemic lupus erythematosus and systemic sclerosis	[59]
rs17079281	Chr6q22.2 *DCBLD1* promoter	C	YY1 (ChIP-qPCR)	*DCBLD1* (ASE, CRISPR/Cas9)	Lung cancer	[16]

Notes: allele-specific binding (ASB), allele-specific expression (ASE), transcription factors (TFs), DNase I hypersensitive sites (DHSs).

**Table 3:** Main features of the most widespread approaches to the search for functional SNPs.

	Approach	GWAS	eQTL Analysis	ASE	ASB
1	Initial association with trait	+	−	−	−
2	Initial association with function	−	+	+	+
3	Causal or in LD	Both	+	++	+++
4	Number of participants	Tens and hundreds of thousands (large cohorts)	Hundreds (modestly sized cohorts)	Few	Few

In row 3, +/++/+++ shows an increase in the bias towards causal.
